# Personal growth initiative: confirmatory factor analysis, gender invariance, and external validity of the Persian version

**DOI:** 10.3389/fpsyg.2025.1576783

**Published:** 2025-07-16

**Authors:** Mojtaba Habibi Asgarabad, Pardis Salehi Yegaei, Parishad Bromandnia, Joseph Ciarrochi, Stefanos Mastrotheodoros, Nora Wiium

**Affiliations:** ^1^Department of Psychology, Norwegian University of Science and Technology, Trondheim, Norway; ^2^Department of Psychology, Faculty of Humanities and Social Sciences, Istinye University, Istanbul, Türkiye; ^3^Health Promotion Research Center, Iran University of Medical Science, Tehran, Iran; ^4^Department of Psychology, Philipps University of Marburg, Marburg, Germany; ^5^Institute for Positive Psychology and Education, Australian Catholic University, North Sydney, NSW, Australia; ^6^Faculty of Social and Behavioral Sciences, Research Center for Adolescent Development, Utrecht University, Utrecht, Netherlands; ^7^Department of Psychology, University of Crete, Crete, Greece; ^8^Department of Psychosocial Science, Faculty of Psychology, University of Bergen, Bergen, Norway

**Keywords:** adolescents, concurrent validity, gender invariance, internalizing and externalizing behavioral problems, personal growth initiative

## Abstract

**Introduction:**

The current cross-sectional research was performed to verify the measurement soundness of the Personal Growth Initiative Scale-II (PGIS-II) regarding reliability, validity, and gender invariance in an Iranian sample.

**Methods:**

In an online survey, 1,453 students (50.8% girls, mean_age_ = 15.48, SD = 0.97) were recruited from several high schools located in Tehran to complete the Persian version of PGIS-II, Youth Self-Report (YSR) of internalizing and externalizing behavior problems, and demographic characteristics.

**Results:**

The original 4-factor structure of PGIS-II demonstrated the best fit in the Confirmatory factor analysis and was invariant across gender. Reliability estimates of this factorial model, including corrected item-total correlation, inter-item correlations, Cronbach’s alpha, Theta, and Omega were good to excellent (e.g., α = 0.86–0.95). Discriminant validity was upheld via the moderate correlation among PGIS-II’s subscales, and through the acceptable levels of average variance extracted. The concurrent validity of the Persian version of PGIS-II and its subscales was supported by their moderate negative correlations with internalizing and externalizing behavioral problems (*r* = −0.20 to −0.42) and their moderate positive correlations with educational performance (*r* = 0.21–0.34). Gender differences emerged, such that boys scored higher on PGIS-II and the subscale of using resources.

**Discussion:**

Overall, the PGIS-II seems suitable for application in the Persian context to capture personal growth initiative. Clinicians and school counselors should devote attention to the personal growth initiative as a key mechanism to prevent adolescents’ behavior problems and improve academic performance.

## 1 Introduction

As one of the six dimensions of well-being and a personal resource for enhancing healthy development essential for proper functioning (Ryff and Keyes, 1995), personal growth is perceived as people’s evaluation regarding the ability to prevail over difficulties they are confronted with in the environment (Robitschek, 2003). Conceptualized based on the personal growth construct, “Personal Growth Initiative” (PGI) was coined by Robitschek (1998) and was defined as being fully aware and actively engaged in self-improvement. Thus, PGI represents a person’s cognitive and behavioral tendency to improve on a spectrum from less to highly competent (Robitschek and Kashubeck, 1999). Increased levels of PGI are usually accompanied by the capacity to recognize conditions that promote personal growth.

To capture the level of PGI, the Personal Growth Initiative Scale (PGIS) was developed (Robitschek, 1999) in English language and was further revised by Robitschek et al. (2012), labeled as the Personal Growth Initiative Scale-II (PGIS-II). PGIS-II reflects a first-level 4-factor structure model, with two cognitive and two behavioral subscales: (i) readiness for change that attributes to the cognitive capability to recognize when a change needs to be done and finding a proper time for change to be applied, (ii) planfulness, the next cognitive sub-scale that shows how prepared a person is to make an appropriate plan on the way of changing, (iii) using resources, the first behavioral subscale that determines the individuals’ ability to identify external resources as well as a support system that can help them to achieve the change successfully, and (iv) intentional behavior, the second behavioral subscale explaining one’s initiation and implementation ability (Robitschek et al., 2012). Theoretically, Bandura’s properties of human agency, intentionality, and planfulness (Bandura, 2001) align with PGI’s subscales of intentional behavior and planfulness. The planfulness subscale is similar to Bandura’s idea of planfulness, highlighting the ability to construct organized strategies for goal achievement. Both frameworks emphasize proactive, self-directed growth as central to human development. Similarly, the intentional behavior subscale reflects Bandura’s concept of intentionality, which involves active commitment to personal goals and purposeful action (Bandura, 2001).

Multiple translations and evaluations of the PGIS II have been conducted across various languages, such as Indian (Bhattacharya and Mehrotra, 2014), Polish (Borowa et al., 2020), Brazilian (Freitas et al., 2018), American (Robitschek et al., 2012), Japanese (Tokuyoshi and Iwasaki, 2014), Turkish (Yalcin and Malkoç, 2013), and Chinese (Yang and Chang, 2014), providing support for the original factorial structure with satisfactory reliability (see Table 1 for more details). With regards to the concurrent validity of the scale among adolescents to date, a few studies showed the predictive role of PGI on posttraumatic stress (Shigemoto et al., 2016), psychological distress (Ayub and Iqbal, 2012; Weigold et al., 2013), psychological well-being (Ayub and Iqbal, 2012; Weigold et al., 2013), and suicidal ideation (Robitschek et al., 2022). The adverse effect of PGIS-II on psychological distress might be explained by the less developed behavioral skills, which lead to high anxiety levels and the use of immature emotion-focused coping strategies (Weigold and Robitschek, 2011). For instance, low PGI predicts difficulty adjusting to new environments, which causes individuals to feel more stressed, anxious, and disappointed throughout their lives (Yakunina et al., 2013; Yakunina et al., 2013). Likewise, PGI was found to have a predictive role in risk-taking behaviors (Jiao et al., 2024). However, prior studies relied on the PGIS-II and its association with adolescent mental health without first testing its construct validity on this population. Indeed, most of the abovementioned studies on the factor structure of PGIS-II have focused on adults. Given its link to adolescent mental health issues, it is crucial to evaluate the PGIS-II’s construct validity via factor analysis and concurrent validity via its relationship with behavior problems to add to the existing evidence.

**Table 1:** Studies validating the psychometric properties of the PGIS-II in different populations.

Authors	Country	Participants	Factor structure	Items	Reliability
Bhattacharya and Mehrotra (2014)	India	Study 1 and 2 (*n* = 89, *M*_age_ = 21) Study 3 (*n* = 234)	Range of factor loadings (EFA; two-factor model) = 0.43–0.77	Awareness of the need for change = 1, 2, 3, 5, 10, 13, and 15 Acting on the awareness = 4, 7, 9, 12, and 14	Total score = 0.81 Awareness of the need for change = 0.77 Acting on the awareness = 0.71
Yalcin and Malkoç (2013)	Turkey	*n* = 279 *M*_age_ (SD) = 20.29 (2.30)	Fit index (CFA; four-factor model model) = CFI = 0.98, RMSEA = 0.06	Readiness for Change = 2, 8, 11, and 16 Planfulness = 1, 3, 5, 10, and 13 Using resources = 6, 12, and 14 Intentional Behavior = 4, 7, 9, and 15	Total score = 0.80 Readiness for Change = 0.83 Planfulness = 0.61 Using resources = 0.62 Intentional Behavior = 0.73
Borowa et al. (2020)	Poland	*n* = 530 *M*_age_ (SD) = 27.83 (12.18)	Fit index (CFA; four-factor model model) = CFI = 0.99, RMSEA = 0.07	Readiness for Change = 8, 11, and 16 Planfulness = 1, 2, 3, 5, 10, and 13 Using resources = 6 and14 Intentional Behavior = 4, 7, 9, 12, and 15	Total score = 0.91 Readiness for Change = 0.86 Planfulness = 0.79 Using resources = 0.81 Intentional Behavior = 0.85
Freitas et al. (2018)	Brazil	*n* = 2,149 *M*_age_ (SD) = 37.91 (10.78)	Range of factor loadings (EFA; four-factor model) = 0.30–0.91	Readiness for Change = 2, 8, 11, and 16 Planfulness = 1, 3, 5, 10, and 13 Using resources = 6, 12, and 14 Intentional Behavior = 4, 7, 9, and 15	Readiness for Change = 0.86 Planfulness = 0.79 Using resources = 0.75 Intentional Behavior = 0.78
Weigold et al. (2014)	United States	*n* = 159 *M*_age_ (SD) = 22.20 (6.76)	Fit index (CFA; four-factor model model) = CFI = 0.92, SRMR = 0.05	Readiness for Change = 2, 8, 11, and 16 Planfulness = 1, 3, 5, 10, and 13 Using resources = 6, 12, and 14 Intentional Behavior = 4, 7, 9, and 15	Total score = 0.90 Readiness for Change = 0.90 Planfulness = 0.92 Using resources = 0.79 Intentional Behavior = 0.89
Shigemoto et al. (2015)	United States [Hispanics (study 1), African Americans (study 2), European Americans (study 3)]	Study 1 [*n* = 218, *M*_age_ (SD) = 32.78 (11.73)] Study 2 [*n* = 129, *M*_age_ (SD) = 32.77 (15.78)] Study 2 [*n* = 552, *M*_age_ (SD) = 34.66 (13.74)]	Fit index (CFA; four-factor model model) = CFI = 0.95, RMSEA = 0.057	Readiness for Change = 2, 8, 11, and 16 Planfulness = 1, 3, 5, 10, and 13 Using resources = 6, 12, and 14 Intentional Behavior = 4, 7, 9, and 15	Study 1 (Total score = 0.91 Readiness for Change = 0.77 Planfulness = 0.81 Using resources = 0.78 Intentional Behavior = 0.82) Study 2 (Total score = 0.88 Readiness for Change = 0.76 Planfulness = 0.75 Using resources = 0.73 Intentional Behavior = 0.80) Study 3 (Total score = 0.92 Readiness for Change = 0.86 Planfulness = 0.87 Using resources = 0.85 Intentional Behavior = 0.86)

N, Sample size; M, Mean; EFA, Exploratory factor analysis; CFA, Confirmatory factor analysis; CFI, Comparative fit index; RMSEA, Root means square error of approximation.

PGI has been deemed a crucial contribution to educational performance. For instance, in the Self-Determination Theory (SDT; Deci and Ryan, 2000), it is proposed that personal growth enhances basic psychological needs satisfaction, realization of individual potentialities, and self-actualization (Robitschek et al., 2012; Ryff, 1989), which are thought to result in successful personal goal fulfillment and academic achievement (Çelik, 2015). Empirical evidence has provided support for this argument. For example, Malik et al. (2013). In a study on 150 Pakistani students, researchers found that two subscales of planfulness and intentional behavior could positively predict academic achievement.

Concerning measurement invariance, the scale must be perceived equally among girls and boys. Only one study conducted a multi-group confirmatory factor analysis on PGIS-II in a Brazilian sample (Freitas et al., 2018) and showed the metric and scalar equivalence of this scale across gender. However, evidence was mixed on gender discrepancies in the level of PGIS-II total score and subscales. While some studies demonstrated that boys score equal to girls in the level of PGIS-II (Robitschek, 1998; Robitschek et al., 2012; Borowa et al., 2020; Yang and Chang, 2014; Weigold et al., 2014), others found lower levels in girls (Kaur and Singh, 2017; Gohlan and Singla, 2016). However, conclusions cannot be drawn based on a few studies, and gender discrepancies for this scale still need to be examined.

Inconsistent societal standards in different cultures are thought to influence PGI manifestation. Cross-cultural variations have been detected in some of the PGIS-II factors in different societies (Borowa et al., 2020). For instance, European Americans, compared to African or Latin Americans, showed a lower score in the mean of PGIS-II subscales (Shigemoto and Robitschek, 2018). Freitas et al. (2018) also found a greater inter-correlation of using resources subscale with other PGIS-II subscales in Brazil, probably due to higher emotional expressiveness among people in Brazilian culture. Robitschek (2003) maintains that although the concept of PGI is similar among various contexts, cultural characteristics decide how an individual pursues growth. Iran is known to have a collectivistic culture (Abbasi et al., 2002), in which cohesion, interdependence, and harmony are praised (Eaton and Louw, 2000). Such cultural values may modify the manifestation of “intentional behavior” and “using resources.” A logical conclusion is that although a collectivistic culture might spur a person to take advantage of group and family support, it prevents them from conflict and independence (Robitschek, 2003; Robitschek et al., 2012). Besides, given that Iranian samples score lower on mental well-being (Joshanloo et al., 2013), the necessity for a Persian version of PGIS-II to quantify personal growth initiative, as an essential well-being factor, across the Iranian population is beneficial. This study uniquely contributes to the literature by culturally adapting the PGIS-II for use in a collectivistic Iranian context, addressing how cultural values may shape the expression of personal growth initiative.

The current study sought to test the psychometric soundness of the Persian PGIS-II with a sample of Iranian youth. Our first objective is to evaluate the theory-derived four-factor construct of the scale in an Iranian sample. Second, the invariance of the perception of the total score and subscales of PGIS-II was evaluated across gender. Third, the reliability was assessed to observe how consistently PGIS-II measures PGI. Fourth, PGIS-II’s discriminant validity was examined by correlating PGIS-II with behavioral problems and educational performance. Finally, the gender differences in the level of PGIS-II total score and the subscales were evaluated. We hypothesized that the total PGIS-II and subscales scores would have negative correlations with internalizing and externalizing problems and positive correlations with educational performance. Beyond psychometric validation, the research emphasizes the scale’s clinical utility in identifying adolescents at risk for behavioral problems and tailoring culturally sensitive interventions.

## 2 Materials and methods

### 2.1 Participants

A total number of 1,453 adolescents in Tehran, aged 14–18, were recruited (50.8% girls; Mean_age_ = 15.48, Standard Deviation (SD) = 0.97). Participants were selected from school class levels 9 (16%), 10 (37.9%), 11 (27.6%), and 12 (18.6%). Concerning parents’ educational background, among fathers, 3.5% had no formal education, 74.7% held a diploma or lower degree, and 21.8% held a college degree. 4.1% of mothers had no formal education, 77.4% held a diploma or less, and 18.7% obtained a college degree. The majority of fathers (95.7%) were employed, while only 3.7 and 0.6% were unemployed or retired, respectively; Conversely, mothers were mainly unemployed (84.4%) with 15.5% employed and 0.1% retired. At the time of the survey, 89.9% of the students lived with both parents, and 8.7% lived with single parents. The remaining participants (1.4%) lived alone or with someone else.

### 2.2 Measurements

#### 2.2.1 Personal growth initiative scale

The 16-item PGIS-II (Robitschek et al., 2012) offers an evaluation of one’s self-improvement ability and growth experiences. PGIS-II has 4 subscales with four items for each subscale: (1) readiness for change (α = 0.87; e.g., “*I figure out what I need to change about myself*”), (2) planfulness (α = 0.90; e.g., “*I know how to set realistic goals to make changes in myself*”), (3) using resources (α = 0.86; e.g., “*I use resources when I try to grow*”), and (4) intentional behavior (α = 0.87; e.g., “*I actively work to improve myself*”). Participants rated how well the items described them using a 6-point Likert scale from 0 = “*strongly disagree*” to 5 = “*strongly agree*.” The items corresponding to each subscale were summed and averaged to calculate the subscale scores. A total score could also be acquired as the average score of subscales (Robitschek et al., 2012).

#### 2.2.2 Youth self-report

YSR was developed by Achenbach and Rescorla (2001) to measure internalizing and externalizing behavior problems for adolescents aged 11–18 years, with subscales of (1) Anxious/depressed (13 items; e.g., “*I have trouble sitting still*”), (2) withdrawal/depressed (8 items; e.g., “*I would rather be alone than with others*”), (3) somatic complaints (10 items; e.g., “r*ashes or other skin problems*”), (4) rule-breaking behavior (15 items; e.g., “*I set fire*”), and (5) aggressive behavior (17 items; e.g., “*I threaten to hurt people*”) are the subscales. Items were rated on a 3-point range (0 = “*not true*,” 1 = “*sometimes/somewhat true*,” and 2 = “*very/often true*”). The YSR’s original factor structure was confirmed in Iranian youth, with acceptable internal consistency, test–retest reliability, inter-rater reliability, and convergent validity (Habibi Asgarabad et al., 2009; Habibi et al., 2009). Internal consistency in this study was excellent for Internalizing (α = 0.92) and Externalizing problems (α = 0.91).

#### 2.2.3 Educational performance

The students’ perception of their educational performance was evaluated using a single item. They were asked to score their performance on a Likert scale from poor (1) to excellent performance (5).

### 2.3 Procedure

The PGIS-II was primarily translated into the Persian language by a group of three bilingual mental health professionals and linguists. The Persian PGIS-II was then back-translated into English. A linguist expert assessed the back translation to ensure that the Persian translation of the PGIS-II is consistent with its original English version. To ascertain any lack of clarity within the Persian items of PGIS-II, a pilot study was initially carried out with a group of 30 volunteer students (50% boys) from one of the participating schools. They were asked to fill in the PGIS-II scale and evaluate PGIS-II items on a range from *not understandable* (0) to *completely understandable* (5). Results on the data collected from these students showed that 97% identified the items as entirely understandable, proving that item change was not required. Results from this preliminary study were not included in the primary analysis.

Using a convenience sampling method, 6 out of 16 invited segregated high schools in Tehran (3 girls’ schools) accepted the opportunity to participate in the study, resulting in a 37.5% school-level response rate. Out of 1,800 students within all participating schools, 1,535 students and their parents consented to participate (an 85.28% response rate). All participants completed the PGIS-II and YSR scales via an online link. In the end, 1,453 students completed all scales correctly and were considered for the final analysis, resulting in a completion rate of 94.7%. All students were informed of the study’s objectives and that participating was optional. In addition, parents of the volunteer students were provided with consent forms, and participants were ensured confidentiality and urged to be as accurate and honest as possible when answering the questions. This study received approval from the ethics board of the Iran University of Medical Sciences (Approval ID = IR. IUMS. REC.1399.1129).

### 2.4 Data analysis

The data screening involved consistency checks, descriptive and graphical analysis, and outlier detection to ensure data accuracy. IBM SPSS Statistics (Version 28) was used to screen data. We found homogeneity in all items of PGIS-II with no missing data (*N* = 1,453). All items met the univariate outlier criteria [−2.00 > Z x > +2.00]. The original data was analyzed without deleting outliers, as recommended by Tabachnick et al. (2007). The normality assumption was assessed, indicating that most items exhibited a mild positive skewness, which was not significant (Gravetter et al., 2020). Using R version 4.1.2 (Revelle, 2017; Team RC, 2013) we evaluated Cronbach’s alpha coefficient and mean inter-item correlation, and following the guidelines for ordinal Likert-type scales (Gadermann et al., 2012; Zumbo et al., 2007), we assessed the equivalent of Cronbach’s alpha coefficient (reliability coefficients of Theta and Omega) derived from the polychoric correlation matrix. According to Cicchetti’s rule of thumb (Cicchetti, 1994), an internal consistency level of 0.70 or higher was considered acceptable.

The confirmatory factor analysis (CFA) was run through Mplus version 8.8 (Muthén and Muthén, 1998–2022), applying the robust maximum likelihood (MLR) to evaluate the hypothesized factor structure of the PGIS-II, as proposed by Robitschek et al. (2012). The robust maximum likelihood (MLR) estimator was employed for the analysis. Although the data were ordinal, additional analyses using WLSMV estimation yielded results comparable to MLR; therefore, MLR was retained for consistency across analyses.

To test the models’ goodness-of-fit, multiple statistical indices and tests were utilized, including the Tucker-Lewis Index (TLI), standardized root means square residual (SRMR), chi-square (*χ*^2^), normed chi-square (*χ*^2^/df), comparative fit index (CFI), root mean square error of approximation (RMSEA), and its 90% confidence interval. To define the suitable values for fit index, we used the recommendations of Bentler and Bonett (1980), Hooper et al. (2008), Hu and Bentler (1999), Loehlin and Beaujean (2016), Mac Callum et al. (1996), and Miles and Shevlin (2007). In line with the approach outlined by Satorra and Bentler (2010), the Bayesian information criterion (BIC) was included to evaluate and compare the fit of competing models, with the model displaying the lower BIC value deemed to provide a better fit. Furthermore, a chi-square difference test was performed using the MLR chi-square to assess the fit between the base and nested models.

To test the measurement invariance across gender, we assessed the configural, metric, and scalar invariances of the best-fitting factor model. Invariance was evaluated by comparing RMSEA values and their 90% confidence intervals, with metric invariance confirmed if RMSEA values fell within each other’s intervals. Changes in CFI, SRMR, and RMSEA were also examined. Measurement invariance required meeting at least two of the following: ∆CFI 0.01, ∆RMSEA ≤ 0.015, and ∆SRMR ≤ 0.03 for factor loading invariance, or ∆SRMR ≤ 0.01 for intercept and residual invariance (Cheung and Rensvold, 1999; Cheung and Rensvold, 2002; Sass et al., 2014).

To implement the multi-group model across gender, we calculated the discrepancies between the chi-squared statistics for the alternative and null models. However, when running the model in Mplus with MLR estimators, a warning message appeared, indicating that the standard chi-squared difference test was not valid for MLR estimators. It is recommended that “DIFFTEST” be selected for nested models and “NESTED” for models with matching degrees of freedom (df). In our case, we ran “NESTED” to verify whether the models are nested, rather than “DIFFTEST,” due to identical “DF” values in the freely estimated model and the models for boys and girls (Asparouhov and Muthén, 2022).

Next, criterion validity was examined by analyzing the association between the PGIS-II and the YSR. Given the non-normality of the data, we used Kendall’s rank correlation coefficient (τb) to assess the relationships of the PGIS-II with measures of behavior problems and educational performance. Effect sizes were categorized as small (0.10), medium (0.30), large (0.50), and very large (0.70), according to Cohen (Cohen, 1988). Average Variance Extracted (AVE) was computed to examine PGIS-II’s discriminant validity.

To capture the mean and SD differences of PGIS-II scores across genders, we applied Multivariate Analysis of Variance (MANOVA) and calculated effect size using Hedge’s g. We categorized effect sizes, following Cohen’s guidelines (Cohen, 1988), as small (< 0.20), medium (0.21–0.50), large (0.51–0.80), and very large (> 0.80).

## 3 Results

### 3.1 Factor structure

The initial model (M1) was designed as a unidimensional structure, with all 16 items loading onto a single common factor reflecting personal growth. This model accounted solely for random measurement error and indicator-specific variance (Gustafsson and Åberg-Bengtsson, 2010). A strong fit of the data to this model would imply limited discriminant validity among the subscales of the psychological instrument. Model two (M2) consisted of a two-factor oblique model with 12 items (excluding items 6, 8, 11, and 16) representing two correlated latent factors reflecting four distinct dimensions of personal growth (Bhattacharya and Mehrotra, 2014). The third model (M3) examined a four-factor oblique model (Borowa et al., 2020). The fourth model (M4) also tested a four-factor oblique model (Freitas et al., 2018; Yalcin and Malkoç, 2013; Weigold et al., 2014; Shigemoto et al., 2015). For a comprehensive overview of the third and fourth models, please refer to Table 1.

### 3.2 Model selection

**Figure 1:**
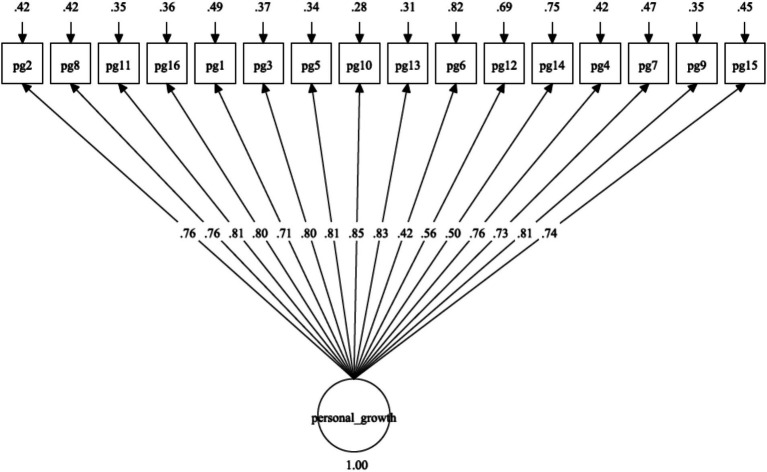
Confirmatory factor analysis of the general factor of PGIS-II.

**Figure 2:**
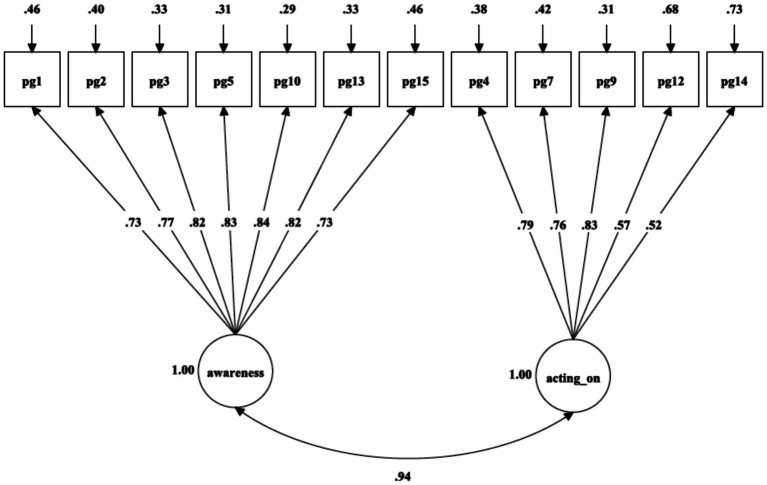
Confirmatory factor analysis of Bhattacharya and Mehrotra’s (2014) two-factor model of PGIS-II.

**Figure 3:**
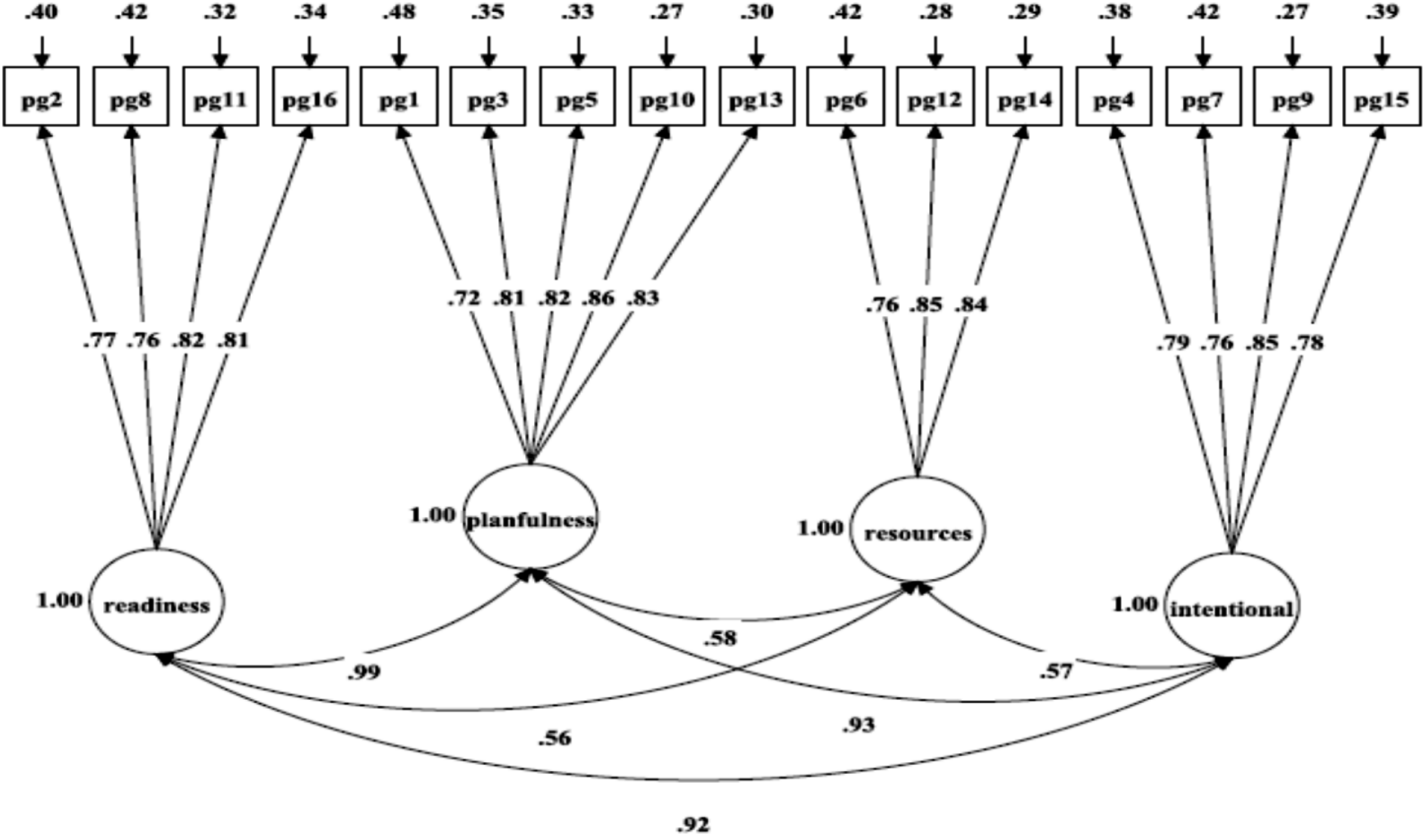
Confirmatory factor analysis of Yalcin and Malkoç (2013), Freitas et al. (2018), Weigold et al. (2014), and Shigemoto’s et al. (2015) four-factor model of PGIS-II.

**Figure 4:**
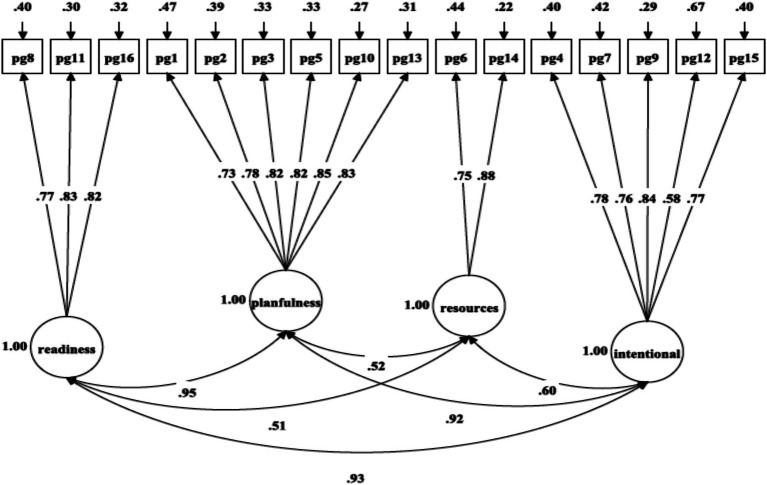
Confirmatory factor analysis of Borowa’s et al. (2020) four-factor model of PGIS-II.

As presented in Table 2, the fit indices for the unidimensional model (Figure 1: χ^2^(104) = 1555.34, *p* = 0.001, RMSEA = 0.095, SRMR = 0.070, CFI = 0.851, TLI = 0.828) and two-factor oblique model (Figure 2: χ^2^(53) = 844.36, *p* = 0.001, RMSEA = 0.099, SRMR = 0.057, CFI = 0.885, TLI = 0.857) failed to satisfy the majority of the fit criteria. Two alternative four-factor oblique models, proposed as prior and theory-derived models, were tested, and their goodness-of-fit was examined, as presented in Table 2. The four-factor oblique model (Table 2 and Figure 3: M4; χ^2^(98) = 647.31, p = 0.001, RMSEA = 0.060, SRMR = 0.034, CFI = 0.944, TLI = 0.931) proposed by Freitas et al. (2018), Shigemoto et al. (2015), Weigold et al. (2014), and Yalcin and Malkoç (2013) demonstrated better fitness than the four-factor oblique model (Table 2 and Figure 4: M3; χ^2^(98) = 1062.97, *p* = 0.001, RMSEA = 0.080, SRMR = 0.056, CFI = 0.901, TLI = 0.879) proposed by Borowa et al. (2020).

**Table 2:** Measurement model and invariance of the PGIS-II across gender in Iranian high school students.

Model	*χ* ^2^	*df*	Scaling	*χ*^2^/df	CFI	TLI	RMSEA	SRMR	Based model	Δ*χ*^2^(df)	ΔCFI	ΔTLI	ΔRMSEA	ΔSRMR
M_1_ = General one-factor	1555.348	104	1.8155		0.851	0.828	0.095 (0.091–0.100)	0.070	-	-				
M_2_ = Short-form two factor (Bhattacharya and Mehrotra, 2014)	844.365	53	1.7808		0.885	0.857	0.099 (0.093–0.105)	0.057	M_1_	712.96 (51)^***^				
M_3_ = Four factor (Borowa et al., 2020)	1062.973	98	1.7975		0.901	0.879	0.080 (0.076–0.086)	0.056	M_1_	432.82 (6)^***^				
M_4_ = Four factor (Freitas et al., 2018; Yalcin and Malkoç, 2013; Weigold et al., 2014; Shigemoto et al., 2015)^†^	647.315	98	1.7866		0.944	0.931	0.060 (0.056–0.065)	0.034	M_1_	728.83 (6)^***^				
Invariance across gender (*n*_boys_ = 765, *n*_girls_ = 767)														
Model boys	303.252	98	1.7417		0.956	0.946	0.052 (0.046–0.059)	0.033	M_4_	0.00000000^††^				
Model girls	488.008	98	1.7103		0.928	0.912	0.072 (0.066–0.078)	0.040	M_4_	0.00000000^††^				
Configural	789.579	196	1.7260		0.941	0.928	0.063 (0.058–0.067)	0.037	–	–				
Metric	821.732	208	1.6946		0.939	0.930	0.062 (0.058–0.067)	0.041	Configural	25.136 (12)^*^	0.002	0.002	0.001	0.004
Scalar	872.356	224	1.6451		0.936	0.931	0.061 (0.057–0.066)	0.045	Metric	67.004 (16)^***^	0.005	0.003	0.002	0.008

χ^2^, Chi-square; df, Degrees of freedom; TLI, Tucker–Lewis index; CFI, Comparative fit index; ABIC, Sample-size adjusted Bayesian information criterion; χ^2^/df, Normal chi-square; Δχ^2^, Difference between minus twice log likelihoods between the full and the nested models; SRMR, Standardized root mean square residual; RMSEA, Root mean square error of approximation; Δ, Differences between parameters of two models. ^†^The final selected model. ^††^Fit function value; nested model check showed that these models are equivalent. ^∗^*p* < 0.05, ^∗∗^*p* < 0.01, ^∗∗∗^*p* < 0.001.

In line with the parsimonious principle (Bollen, 1989), the fit indices of the four-factor first-order model (M4) were compared with those of the competing model. A Nesting and Equivalence Testing (NET) approach was used to identify the best parsimonious model, applying MLR as an estimation method in Mplus 8.8 (Satorra and Bentler, 2010; Asparouhov and Muthén, 2022). Finally, the comparison of M3 with M4 as nested competitive models showed that these models were not equivalent/nested [Fit Function Value (FFV) = 0.11806742]. Consequently, the four-factor oblique model (M4 and Figure 3) was identified as the optimal and most parsimonious model, as it exhibited a lower chi-square value and better goodness-of-fit than M3.

**Figure 5:**
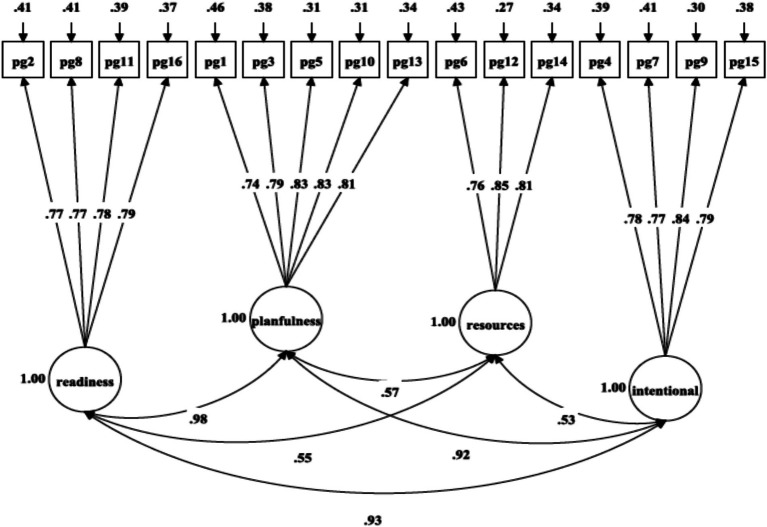
Confirmatory factor analysis of Yalcin and Malkoç (2013), Freitas et al. (2018), Weigold et al. (2014), and Shigemoto’s (2015) four-factor model of PGIS-II for boys.

**Figure 6:**
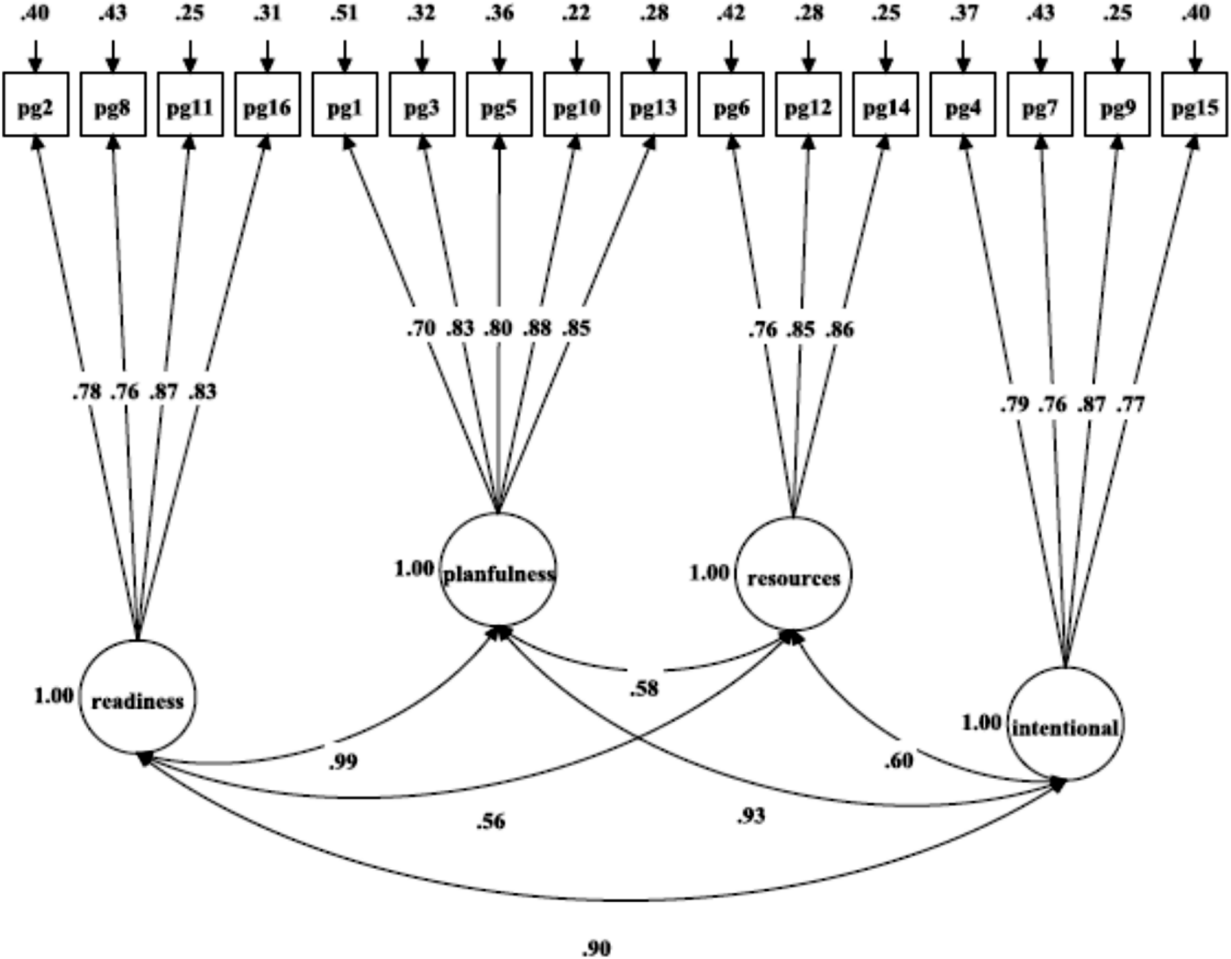
Confirmatory factor analysis of Yalcin and Malkoç (2013), Freitas et al. (2018), Weigold et al. (2014), and Shigemoto’s et al. (2015) four-factor model of PGIS-II for girls.

A multi-group CFA analysis was run to investigate the equivalence of PGIS-II measurement across gender in high school-aged boys and girls. Initially, the CFA analysis was performed both on the entire sample and separately for boys and girls to establish a satisfactory baseline model from a parsimonious and meaningful perspective (Werts et al., 1976). The selected model (Table 2: M4 and Figure 3) was run in both boys (Table 2 and Figure 5: χ^2^(98) = 303.25, *p* = 0.001, RMSEA = 0.052, SRMR = 0.033, CFI = 0.956, TLI = 0.946) and girls (Table 2 and Figure 6: χ^2^(98) = 488.01, *p* = 0.001, RMSEA = 0.072, SRMR = 0.040, CFI = 0.928, TLI = 0.912) separately (Werts et al., 1976). The evaluation of configural invariance, along with weak and substantial factorial equivalence, was carried out by examining the patterns of fixed and free parameters, factor loadings, and item intercepts/means/thresholds (Cheung and Rensvold, 2002; Byrne et al., 1989; Meredith, 1993).

Minimal modifications in the model fit indices supported the establishment of configural invariance for the four-factor oblique model across gender. As shown in Table 2, the results revealed equivalent form, equal factor loadings, and non-equal item intercepts across gender, indicating that the same construct is being measured for both genders. Finally, comparing the metric model with the configural model (ΔCFI = 0.002, ΔRMSEA = 0.002, ΔSRMR = 0.001) and the scalar model with the metric model (ΔCFI = 0.005, ΔRMSEA = 0.003, ΔSRMR = 0.002) suggested that the most optimal and parsimonious across gender was the four-factor oblique model (Table 2: M4).

### 3.3 Internal reliability

Table 3 provides an overview of various statistical properties of the PGIS-II subscales, including descriptive statistics, reliability coefficients (Cronbach’s alpha, ordinal Theta, and Omega), and the corrected item-total correlation. In general, most items across the four subscales demonstrated moderate positive intercorrelations. The corrected item-total correlations varied between 0.31 and 0.81 for subscale items and between 0.27 and 0.78 for total scale items, indicating consistent internal reliability across the measure. Moreover, the inter-item correlations were reasonably moderate, with mean values of 0.49, 0.50, 0.54, 0.54, and 0.57 for the total scale, the readiness for change, planfulness, using resources, and intentional behavior subscales, respectively. Results of Cronbach’s alpha (0.86–0.95), Theta (ordinal alpha) (0.88–0.96), and Omega (0.86–0.96) reliabilities indicated excellent internal consistency of PGIS-II (Table 3).

**Table 3:** Descriptive statistics and correlation coefficients of personal growth with behavior problems and educational performance.

	1	2	3	4	YSR-IN	YSR-A/D	YSR-W/D	YSR-SC	YSR-EX	YSR-RBB	YSR-AB	YSR-Total	EP	Mean (SD)			Theta	Omega	α	AVE
														Total	Male	Female				
1. Readiness for change	–	0.88^**^	0.48^**^	0.80^**^	−0.33^**^	−0.30^**^	−0.31^**^	−0.27^**^	−0.31^**^	−0.26^**^	−0.31^**^	−0.35^**^	0.31^**^	14.09 (4.34)	14.19 (4.19)	13.94 (4.40)	0.90	0.87	0.87	0.63
2. Planfulness		–	0.49^**^	0.82^**^	−0.36^*^	−0.33^**^	−^.^36^**^	−^.^29^**^	−^.^34^**^	−0.29^**^	−0.34^*^	−0.39^*^	0.34^**^	17.28 (5.64)	17.43 (5.47)	17.16 (5.76)	0.93	0.90	0.90	0.65
3. Using resources			–	0.49^**^	−0.33^**^	−0.24^**^	−0.36^**^	−0.28^**^	−0.27^**^	−0.20^**^	−0.29^*^	−0.33^**^	0.21^**^	8.69 (3.89)	9.07 (3.71)	8.32 (4.02)	0.88	0.86	0.86	0.68
4. Intentional behavior				–	−0.33^**^	−0.28^**^	−0.34^**^	−0.27^**^	−0.34^**^	−0.29^**^	−0.34^**^	−0.37^**^	0.31^**^	15.05 (4.25)	15.22 (4.13)	14.90 (4.32)	0.90	0.87	0.87	0.63
PGIS-II	0.92^**^	0.94^**^	0.68^**^	0.90^**^	−0.39^**^	−0.33^**^	−0.40^**^	−0.32^**^	−0.37^**^	−0.30^**^	−0.37^**^	−0.42^**^	0.34^**^	55.09 (15.81)	55.85 (15.29)	54.33 (16.29)	0.96	0.96	0.95	0.65

α, Alpha; SD, Standard deviation; YSR, Youth Self-Report of behavior problems; IN, Internalizing problems; A/D, Anxiety/depression; W/D, Withdrawal/depression; SC, Somatic complaints; EX, Externalizing problems; RBB, Rule-breaking behavior; AB, Aggressive behavior; EP, Educational performance; AVE, The concept of average variance extracted (AVE) is a tool for assessing the amount of variance in a construct that is captured by its indicators, relative to the variance that arises from measurement errors. ^∗^*p* < 0.05, ^∗∗^*p* < 0.01.

### 3.4 Discriminant and criterion validity of PGIS-II

Table 3 indicates that the PGIS-II subscales exhibited acceptable levels of AVE for discriminant validity, with scores ranging from 0.63 to 0.68. Similarly, the overall scale also demonstrated a satisfactory level of AVE at 0.65.

Table 3 displays the correlations among the PGIS-II’s subscales, which range from 0.48 (for the relationship between using resources and readiness for change) to 0.88 (for the relationship between readiness for change and planfulness). Regarding concurrent validity, the associations between the PGIS-II and the Youth Self-Report (YSR) of internalizing and externalizing behavioral problems were found to be significant, as indicated in Table 3. The PGIS-II displayed a moderate negative correlation with behavioral problems, with correlations ranging from −0.20 to −0.42. A correlation analysis revealed that educational performance was positively associated with the PGIS-II total score (*r* = 0.34) and its subscales (*r* = 0.21–0.34).

### 3.5 Gender differences and personal growth

Table 3 displays the mean scores (M) and SD for the PGIS-II and its subscales, broken down by gender (boys and girls). Boys scored significantly higher on their total PGIS-II level [*t* (1453) = 1.98, *p* = 0.048] than girls. Through MANOVA, significant gender-related differences on the subscales of PGIS-II [*F* (4, 1,527) = 3.99, *p* = 0.003, partial *Eta* squared = 0.010] were revealed, as outlined in Table 3. Further analysis of between-subjects effects indicated that boys had significantly higher scores than girls on the using resources subscale [*F* (1, 1,531) = 14.55, *p* < 0.001, partial *η*^2^ = 0.009], compared to girls. However, the findings of ANOVA analyses suggested no significant gender difference in terms of readiness for change [*F* (1, 1,531) = 1.28, *p* = 0.257, partial *η*^2^ = 0.001], planfulness [*F* (1, 1,531) = 0.90, *p* = 0.343, partial *η*^2^ = 0.001], and intentional behavior scores [*F* (1, 1,531) = 2.10, *p* = 0.147, partial *η*^2^ = 0.001]. These results suggest that, except for the gender difference observed in using resources, boys and girls did not differ on the other PGIS-II subscales.

## 4 Discussion

Using an Iranian sample, this study examined the preliminary psychometric properties of the Persian version of the PGIS-II. Our findings revealed that the original four-factor model of the PGIS-II had satisfactory fit indices. Subsequently, the significant correlation of the PGIS-II with internalizing and externalizing behavioral problems and educational performance supported the initial evidence of the discriminant validity of the PGIS-II in Iran.

The four-factor structure best fits our Iranian sample, with adequate standardized factor loadings of items (>0.3) on the four subscales of readiness for change, planfulness, using resources, and intentional behavior. This finding was consistent with the original factorial model (Robitschek et al., 2012) and the CFA results in adults found in Poland (Borowa et al., 2020), Brazil (Freitas et al., 2018), Japan (Tokuyoshi and Iwasaki, 2014), Turkey (Yalcin and Malkoç, 2013), and China (Yang and Chang, 2014).

Reliability coefficients of Cronbach’s alpha, Omega, and ordinal Theta confirmed excellent internal consistency of the four PGIS-II dimensions. The reliability was also supported by moderate means of inter-item correlations (0.49–0.57), given that strong relationships indicate the sameness of items’ content, and weak correlations indicate the absence of any similarity between items (Hair et al., 2006). The corrected item-total correlation values for the total score and dimensions showed moderate to good reliability (Ferketich, 1991). These results generally showed acceptable to excellent reliability and are compatible with previous studies (e.g., Yang and Chang, 2014).

It might be misleading to make conclusions on gender differences prior to determining measurement invariance across gender (Vandenberg Vandenberg and Lance, 2000; Whisman et al., 2013). Without invariance testing, it will not be clear if the gender dissimilarities in the mean of PGIS-II are due to genuine gender-specific characteristics of the latent construct or stem from the conceptually different ways boys and girls interpret the items (structural differences). Results of the multi-group CFA showed equality in fixed and free parameters, similarity in factor loading patterns, and equivalency in the indicator means in both genders. These findings align with the findings of Freitas et al. (2018) and suggest that both boys and girls perceive the PGIS-II structure consistently and have similar understandings of the scale’s items. Therefore, PGIS-II might be studied equally across genders (Cheung and Rensvold, 2002; Meredith, 1993).

Inter-correlation between subscales, as an indicator of discriminant validity, revealed moderate to high coefficients, with the link of planfulness and readiness for change being the strongest, perhaps because they both are cognitive aspects influenced by cognitive abilities. Of note, in line with the findings of Robitschek et al. (2012), using resources showed the weakest correlations with other subscales and yielded the lowest means. As Robitschek et al. (2012) suggested, this subscale captures the external processes, while other subscales involve the internal and independent processes of intentional growth. This result, however, is in contrast with Robitschek’s assumption (Robitschek et al., 2012) the mean of using resources and its link with other PGIS-II subscales would be higher in countries with collectivistic cultures. Despite the great force of collectivistic values that urge inter-dependence, our correlation patterns suggested that using resources might not be necessary or central, to put it mildly, in the procedure of intentional personal growth among Iranian adolescents.

Moderate negative correlations between externalizing and internalizing problem subscales (anxious/depressed, withdrawal/depressed, somatization, rule-breaking, and aggressive behavior) and PGIS-II were observed, further confirming the PGIS-II’s discriminant validity. High scores on all PGI’s subscales have been shown to be related to easier recognition of chances for future beneficial personal growth (Robitschek and Kashubeck, 1999), and those with high PGI levels are better at handling stress and adjusting to new conditions (Robitschek et al., 2012; Weigold et al., 2013; Loo et al., 2014). In contrast, those with poorer skills in PGI indicators are believed to have difficulty adapting to a new environment (de Freitas et al., 2016), which leads to higher anxiety and stress (Yakunina et al., 2013; Yakunina et al., 2013; Shigemoto et al., 2015). Based on our results, as the first study to document the link between PGI and externalizing problems, they also seem to manifest their poor life satisfaction and maladjustment with aggressive and rule-breaking behaviors.

The study found that all subscales of PGIS-II exhibited acceptable discriminant validity with an AVE higher than 0.5. This aligns with previous research that recommends a minimum AVE of 0.5 for satisfactory discriminant validity, while an AVE below 0.5 is considered questionable as it suggests that measurement error accounts for more variance than the construct itself (Fornell and Larcker, 1981; Henseler et al., 2015).

Our findings have also demonstrated a significant positive association between educational performance and PGI’s total score and subscales, with planfulness emerging as the strongest correlation among subscales. These findings were consistent with Malik’s study (Malik et al., 2013) that found academic achievement had a positive link with intentional behavior and planfulness, while only planfulness significantly predicted academic achievement. It was an expected finding owing to the emphasis on theoretical approaches (e.g., SDT; Deci and Ryan, 2000) on the importance of PGI in self-actualization and exploitation of faculties, which are believed to lead to better academic performance (Çelik, 2015).

Distinct patterns for girls and boys were found in the level of the PGIS-II total score, with boys scoring notably higher. In the subsequent tests of MANOVA and ANOVA, this pattern was also observed for the subscale of using resources, but not in readiness for change, planfulness, and intentional behavior. This contrasts with previous evidence demonstrating that boys score equal to (Robitschek, 1998; Robitschek et al., 2012; Borowa et al., 2020; Yang and Chang, 2014; Weigold et al., 2014) or lower than girls (Kaur and Singh, 2017; Gohlan and Singla, 2016) in the level of PGI. It is particularly a surprising finding that Iranian boys, compared to girls, have a higher tendency to use environmental resources to foster their growth, since it is expected in a traditional society that the dominant masculine stereotypes hamper males from seeking help (Addis and Mahalik, 2003). Instead, this result may be observed due to boys’ perception of the items’ content. Indeed, items on PGIS-II inquire about “resources” –and not “help” (Robitschek et al., 2012); Therefore, boys’ fear of dependence or self-stigma is less likely to be activated in this case (Addis and Mahalik, 2003; Hammer and Vogel, 2010).

### 4.1 Limitations and future directions

Methodological limitations of the present study should be noted. Primarily, we used the convenient sampling method; hence, the results might be skewed by only including those who want to engage in this survey actively. The application of self-report questionnaires is the second limitation of this study. Thus, any conclusions based on the current results should be drawn cautiously. In addition, educational performance was measured by a single item, which may lead to poor content validity as it cannot capture multiple aspects of the construct (e.g., academic achievement, cognitive skills, motivation, engagement) and might reflect subjective interpretations or biases, limiting its objectivity and generalizability. Furthermore, the existing understanding of PGIS-II can be strengthened through future studies with different research designs and more systematic sampling methods. Further studies should also use a longitudinal design to study PGIS-II psychometrics in healthy and clinical samples. Using this design would also help us understand whether personal growth initiative is the predisposing factor of behavior maladjustment, or if the link is bidirectional. Prospective studies also provide insight into the underlying mechanisms through which PGI may lead to academic achievement or prevent engagement in externalizing problems. The last limitation is the need for studies with heterogeneous populations across all ages, given that different age groups may show different personal growth trends.

## 5 Conclusion

The study’s primary purpose was to support the validity and reliability of the Iranian version of the PGIS-II. Our findings demonstrated good to excellent reliability estimates in the total score and subscales of the PGIS-II. Significant correlations of PGIS-II with behavioral problems were found, supporting the preliminary discriminant validity of this scale in Iran. In conclusion, PGIS-II demonstrates potential for psychometric soundness for use with the Iranian youth. By linking PGIS-II scores to educational outcomes and mental health indicators, the study suggests promising but tentative applicability for school counselors and mental health professionals working with youth in non-Western settings.

## Data Availability

The raw data supporting the conclusions of this article will be made available by the authors, without undue reservation.
